# Mechanism of polypurine tract primer generation by HIV-1 reverse transcriptase

**DOI:** 10.1074/jbc.M117.798256

**Published:** 2017-11-09

**Authors:** Małgorzata Figiel, Miroslav Krepl, Sangwoo Park, Jarosław Poznański, Krzysztof Skowronek, Agnieszka Gołąb, Taekjip Ha, Jiří Šponer, Marcin Nowotny

**Affiliations:** From the ‡ Laboratory of Protein Structure and; ‡‡ Biophysics Core Facility, International Institute of Molecular and Cell Biology, 02-109 Warsaw, Poland,; § Institute of Biophysics of the Czech Academy of Sciences, 612 65 Brno, Czech Republic,; ¶ Regional Centre of Advanced Technologies and Materials, Department of Physical Chemistry, Faculty of Science, Palacky University Olomouc, 771 46 Olomouc, Czech Republic,; Departments of ‖ Biophysics and Biophysical Chemistry,; ¶¶ Biophysics, and; ‖‖ Biomedical Engineering, The Johns Hopkins University, Baltimore, Maryland 21205,; ** Institute of Biochemistry and Biophysics, Polish Academy of Sciences, 02-106 Warsaw, Poland, and; §§ Howard Hughes Medical Institute, Baltimore, Maryland 21205

**Keywords:** cysteine-mediated cross-linking, human immunodeficiency virus (HIV), molecular dynamics, nucleic acid structure, protein-nucleic acid interaction, reverse transcriptase, ribonuclease H

## Abstract

HIV-1 reverse transcriptase (RT) possesses both DNA polymerase activity and RNase H activity that act in concert to convert single-stranded RNA of the viral genome to double-stranded DNA that is then integrated into the DNA of the infected cell. Reverse transcriptase–catalyzed reverse transcription critically relies on the proper generation of a polypurine tract (PPT) primer. However, the mechanism of PPT primer generation and the features of the PPT sequence that are critical for its recognition by HIV-1 RT remain unclear. Here, we used a chemical cross-linking method together with molecular dynamics simulations and single-molecule assays to study the mechanism of PPT primer generation. We found that the PPT was specifically and properly recognized within covalently tethered HIV-1 RT–nucleic acid complexes. These findings indicated that recognition of the PPT occurs within a stable catalytic complex after its formation. We found that this unique recognition is based on two complementary elements that rely on the PPT sequence: RNase H sequence preference and incompatibility of the poly(rA/dT) tract of the PPT with the nucleic acid conformation that is required for RNase H cleavage. The latter results from rigidity of the poly(rA/dT) tract and leads to base-pair slippage of this sequence upon deformation into a catalytically relevant geometry. In summary, our results reveal an unexpected mechanism of PPT primer generation based on specific dynamic properties of the poly(rA/dT) segment and help advance our understanding of the mechanisms in viral RNA reverse transcription.

## Introduction

Human immunodeficiency virus type 1 (HIV-1)^3^ is the causative agent of acquired immunodeficiency syndrome. It is a retrovirus that encodes its genetic information in the form of single-stranded RNA, which must be converted to double-stranded DNA (dsDNA) for integration into the genome of the infected cell (1). This process, termed reverse transcription, is a multistep reaction that, despite its complexity, is catalyzed by a single enzyme: reverse transcriptase (RT). Reverse transcriptases possess DNA polymerase activity and RNase H nucleolytic activity (2). Polymerase activity is used to synthesize the DNA strand on an RNA or DNA template, and RNase H degrades the RNA strand of RNA/DNA hybrid intermediates of the reaction. The two enzymatic activities are coordinated and coupled. For example, RNase H cleavage sites are located ∼18 bp away from the polymerase active site (3, 4). Moreover, the available crystal structures of HIV-1 RT reveal conformational changes in the RNA/DNA as an important element of the mechanism of HIV-1 RT. Two nucleic acid conformations have been observed. In each of these conformations, the substrate interacts with only one active site: DNA polymerase (polymerase mode) or RNase H (RNase H mode) (4–6). Recently, it has been shown biochemically that another conformation exists in which the substrate interacts with both active sites (7, 8). Such conformation was termed “simultaneous mode,” and it was found to involve untwisting of the double helix and narrowing of the minor groove in the region of the substrate that interacts with the RNase H domain. HIV-1 RT functions as a heterodimer that consists of two subunits. p66 is the catalytic subunit with the polymerase and RNase H active sites, and p51 (a shorter version of p66) plays a structural role.

Retroviral RTs, including the enzyme from HIV-1, use a tRNA of the infected cell to prime the synthesis of the first DNA strand (9). An 18-nt region from the 3′ terminus of tRNA hybridizes with the primer-binding site of the viral RNA, which is located close to the 5′ end of the viral genome. A relatively short DNA fragment that comprises an “R” sequence is then synthesized (termed (−)-DNA) from the primer-binding site to the 5′ end of the RNA. During and after DNA synthesis, the complementary RNA strand is degraded by RNase H activity, which liberates the DNA to hybridize with another copy of the R sequence that is located in the 3′ portion of the viral RNA in a “strand transfer” step. Subsequent DNA polymerization leads to the synthesis of the full-length (−)-DNA strand while the RNase H continues to hydrolyze the RNA. Two copies (3′ and central) of an important region of the genome, termed the polypurine tract (PPT), are refractory to RNase H cleavage. They are left intact so they can be used as primers in the synthesis of the second DNA strand, termed (+)-DNA (10, 11). After synthesis of the first fragment of (+)-DNA, the second strand transfer event occurs in which (+)-DNA hybridizes with the 3′ end of (−)-DNA. (+)-DNA is further elongated to the central PPT where polymerization displaces the DNA all the way to the terminator sequence. The resulting overhang is removed by cellular enzymes. The dsDNA is integrated into the genome by retroviral integrase (12).

The complexity of reverse transcription is likely derived from the fact that DNA synthesis requires priming. HIV-1 RT can efficiently extend only two types of RNA primers: tRNA for (−)-DNA synthesis and a PPT primer for (+)-DNA polymerization (10, 13–16). Single-molecule experiments have shown that HIV-1 RT can adopt two orientations when binding a primer hybridized to a longer DNA template (16). RNA primers were bound with an orientation that positions the RNase H domain for cleavage of the RNA strand but does not allow its extension by the polymerase. On DNA primers, the orientation of the enzyme was flipped, thus favoring extension of the 3′ end of the primer. The PPT RNA primer was unique because the RNA/DNA hybrid it formed could be bound by the enzyme in both orientations, the one compatible with RNase H cleavage and the one conducive to polymerization. This explained why the PPT can be extended and serve to initiate the synthesis of the (+)-DNA strand, thus playing a key role in HIV-1 proliferation (11). Generation, extension, and removal of the 3′ PPT create one of the ends of the final dsDNA that is recognized by HIV-1 integrase and essential for incorporating viral genetic information into the genome of the host (17). Therefore, the PPT plays a critical role in the HIV-1 replication cycle.

Polypurine tracts comprise 15 ribonucleotides, including a stretch of eight adenines with a single intervening guanine and a stretch of six guanines. In viral RNA, the PPT is located between a tract of five uridines (U-tract) and a sequence termed U3 (10, 11). Generation of the PPT primer relies on both protection of the body of the PPT from RNase H hydrolysis and the introduction of specific cuts at its termini (within the U-tract and at the PPT-U3 junction) (10, 11). However, the mechanism of this process is unclear. The particular features of the PPT that are used for its recognition are unknown. As demonstrated by nuclear magnetic resonance, PPT RNA/DNA adopts an essentially regular, primarily A-form duplex structure with no evidence of unpairing or mispairing (18). Moreover, the PPT is efficiently cleaved by cellular RNase H1 (10) or isolated retroviral RNase H (19), so it is not inherently resistant to RNase H cleavage. In fact, it is unclear whether the specific properties of the PPT are detected within the RT-nucleic acid complex or at the stage of its binding, leading to a particular register of the PPT interaction with the RT.

In the present study, we used a unique approach that combined enzymatic studies of chemically cross-linked RT-substrate complexes with single-molecule experiments and molecular dynamics (MD) simulations to study PPT generation. We found that the PPT sequence was recognized within the stable HIV-1 RT–substrate complex and not at the stage of the complex formation. This recognition was based on two elements: RNase H cleavage sequence preference and incompatibility of the poly(rA/dT) tract of the PPT with the conformation that is required for RNase H cleavage. The latter was the dominant mechanism that resulted from higher rigidity of the poly(rA/dT) sequence and its slippage upon deformation. Our results revealed an unexpected mechanism of PPT generation that utilizes a unique indirect readout of the homopolymeric nucleic acid sequence that relies on specific dynamic properties of the poly(rA/dT) segment.

## Results

### Processing of PPT substrates within cross-linked complexes

To study PPT recognition by HIV-1 RT, we prepared chemically cross-linked complexes of the protein with RNA/DNA hybrid substrates. This approach allowed us to monitor cleavage events independently of the substrate binding step and at defined sites in the substrates. The cross-linking reaction involved the formation of a disulfide bond between a cysteine residue that was introduced into the protein sequence and a thiol group that was introduced into the base of the DNA strand of the substrate (Fig. S1) (20). DNA modification was performed by introducing 2-fluoroinosine into the DNA oligonucleotide followed by reaction with cystamine, whose amine group substituted the fluorine atom (see “Experimental procedures”). We used a well established tethering protocol with a Q258C variant of HIV-1 RT and a hybrid substrate that was modified on the sixth DNA base from the 3′ end (4, 6). This approach is compatible with all modes: polymerase, RNase H, and simultaneous (8), and captures active configurations of HIV-1 RT complexes as demonstrated by corresponding crystal structures (4, 6).

**Figure 1.**
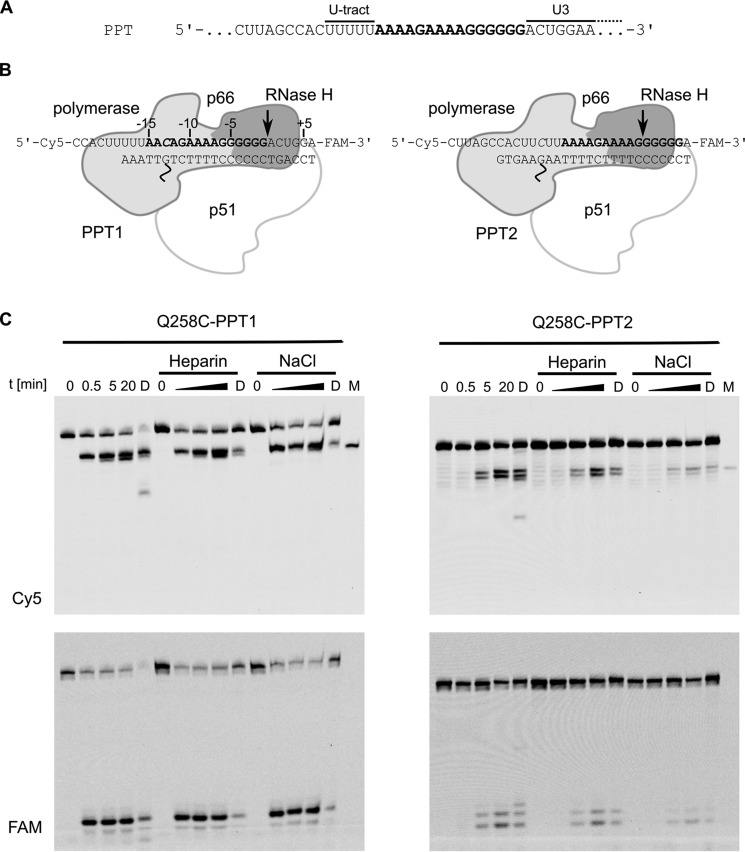
**RNase H cleavage in cross-linked complexes of HIV-1 RT with substrates that comprise the PPT sequence.** *A*, sequence of HIV-1 RNA region that contains the PPT (marked in *bold*). The upstream U-tract and downstream U3 element are indicated. *B*, schematic of the cross-linked complexes of PPT1 and PPT2 hybrid substrates. HIV-1 RT is shown in schematic representation (*light gray* for polymerase domain, *dark gray* for RNase H domain, and *white* for p51). The cross-links are marked with *wavy lines*. The positions of terminal fluorescent dyes (Cy5 and fluorescein (*FAM*)) in the RNA strands are indicated. The expected positions of the RNase H active site and cleavage in the cross-linked complex are indicated with *arrows*. The sequence alteration that is required to introduce thiol-modified guanine in the DNA strand is indicated in *italics*. Numbering of the substrate residues relative to the scissile phosphate (used in all of the analyses) is shown in the *left panel. C*, RNase H cleavage within cross-linked complexes with PPT substrates. Reaction products were separated in urea-polyacrylamide gels and visualized with Cy5 (*upper panels*) or fluorescein (*FAM*) (*lower panels*) fluorescence. *M*, marker (Cy5-labeled 24-mer RNA corresponding to the product of the cleavage 18 nt from the 3′ end of the primer); *D*, reaction in the presence of DTT.

We prepared RNA/DNA hybrids with a double-stranded region of 24 bp and a 6-nt 5′ overhang in the RNA strand. The overhang was introduced to promote the binding of the 3′ end of the DNA primer at the polymerase active site, thus positioning the substrate for cross-linking. The RNA strand contained terminal fluorophores to visualize the cleavage products. Generation of the PPT involves protection of its body from RNase H cleavage and introduction of cuts at its termini. To monitor both elements, we used substrates with sequences that corresponded to the sequence of the PPT in two registers (Fig. 1, *A* and *B*). When the first substrate (PPT1) was cross-linked with HIV-1 RT, the RNase H active site was positioned at the proper cleavage site at the PPT-U3 junction. In the second sequence (PPT2), the register was shifted, positioning the RNase H active site in the middle of the PPT sequence within the G-tract where cleavage should not occur.

Cross-linked complexes were purified (Fig. S2) and used in RNase H cleavage assays, an approach that we developed previously (8). To ensure that the observed cleavage events resulted only from the reaction that was catalyzed by covalently bound complexes, we used heparin as a substrate competitor (trap) or a high NaCl concentration, which prevents protein-RNA/DNA interactions (8). We performed time-course experiments for RNase H cleavage within cross-linked complexes for times between 30 s and 20 min for complexes alone and in the presence of 3 mg/ml heparin or 0.5 m NaCl. RNase H cleavage occurred at the expected position, 18 nt from the polymerase active site (Fig. 1*C*). Additional cuts 17 nt from the polymerase active site were also observed, which we interpreted to result from conformational flexibility of the HIV-1 RT–substrate complex (see “Discussion”). Upon pretreatment of the complexes with 20 mm DTT, which caused breakage of the disulfide cross-link in a fraction of the complexes, new cleavage products were observed (Fig. 1*C*, *lanes* marked “*D*”), resulting from the free enzyme that dissociated from the substrate and bound it with a different register. However, in the presence of high salt concentration or heparin, DTT-pretreated reactions were inhibited, and off-register cleavages were completely eliminated, confirming the validity of our experimental approach. Densitometric quantification of the reaction products in each sample (Fig. S3) demonstrated that PPT1 reactions without heparin and with low NaCl concentration proceeded to a similar extent for the cross-linked complex and for the complex in which the cross-link was broken by the addition of DTT.

The reactions with PPT2 substrates were markedly less efficient than with PPT1, demonstrating that the body of the PPT sequence was refractory to cleavage within the covalently tethered protein-nucleic acid complex. This important result indicated that PPT recognition occurs within a stable/catalytic HIV-1 RT–nucleic acid complex that can form either by association between the substrate and the protein or by stopping the sliding of the enzyme on the RNA/DNA duplex (21). To further verify that preferential binding of the PPT sequence in a particular register does not contribute to the mechanism of PPT generation, we measured the dissociation constants for the interactions between the PPT1 and PPT2 substrates and wild-type HIV-1 RT (Fig. S4). Both values were similar (2.6 nm for PPT1 and 3.7 nm for PPT2), indicating that the enzyme can bind the PPT sequence in both registers equally well. Collectively, these results showed that generation of the PPT does not rely on the binding of RNA/DNA in a particular register but rather depends on events that occur after the formation of a stable protein-nucleic acid complex.

### Effect of sequence motifs on cleavage efficiency by the RNase H domain

**Figure 2.**
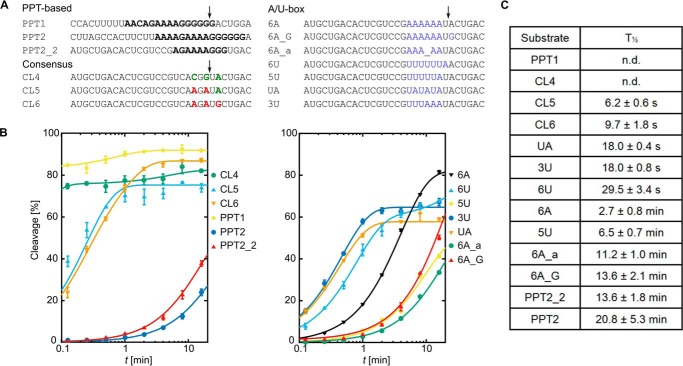
**Effect of substrate sequence on RNase H cleavage within cross-linked complexes.** *A*, substrates used for the analysis of PPT cleavage specificity and sequence determinants of cleavage. Only RNA strands of the hybrid substrates are shown for clarity. All of the sequences are presented from 5′ to 3′. PPT segments are marked in *bold*. For substrates CL4, CL5, and CL6, the preferred residues at the key consensus positions are shown in *green*, and the non-preferred residues are shown in *red*. For A/U-box substrates, the sequences that were replaced in the CL4 substrate are shown in *blue*. The expected cleavage sites are indicated with *arrows. B*, cleavage rates of the substrates listed in *A* within cross-linked complexes. Error bars represent S.D. of three independent measurements. Lines represent the result of global fitting of the data using a pseudo-zero-order reaction. *C*, *t*_½_ values calculated from global fitting of the data. Detailed results with statistical analyses are shown in Table S1, and the global fitting is shown in Fig. S6. *n.d.*, not determined.

To further explore the mechanism that underlies PPT generation, we performed time-course experiments for various substrates in the presence of heparin and with additional time points within the 7.5-s to 16-min range for kinetic analysis. The products of the reaction within the cross-linked complexes were resolved on acrylamide gels and visualized by fluorescence. For each sample, the amount of the cleavage products was expressed as their fluorescence signal divided by the fluorescence of all bands in the lane. This normalized the measurement and removed the effect of any inaccuracies in the quantification of the amount of each complex and gel loading and/or differences in fluorescent properties of the various substrates we used. The extent of the reaction at each time point was fit to a pseudo-zero-order reaction model. PPT1 was cleaved too rapidly for the half-life (*t*_½_) to be reliably determined, but it was estimated to be less than 3 s. For PPT2, *t*_½_ was 20.8 ± 5.3 min, confirming that the PPT in this register was a poor substrate (Fig. 2, Figs. S5 and S6, and Table S1).

The HIV-1 RNase H domain has been reported to have substrate sequence preference for cleavage (22–26). The following consensus in the vicinity of the cleavage site was identified: A or U for position +1; G or C for position −2; and C, G, or U for position −4. In all of our analyses, we used nucleotide numbering relative to the preferred RNase H cleavage site 18 nt from the 3′ end of the DNA primer (Fig. 1*B*). The RNase H consensus was perfectly matched for the PPT1 substrate, but PPT2 contained non-preferred residues in the relevant positions. Therefore, to test the contribution of RNase H sequence preference to PPT recognition, we designed three substrates with a random sequence but specific residues in consensus positions +1, −2, and −4 (Fig. 2*A*). CL4 had a perfect match with the consensus and was cleaved too rapidly for reliable *t*_½_ determination. CL5 had two non-matching residues and was cleaved with *t*_½_ = 6.2 ± 0.6 s. CL6 had three non-matching residues and was cleaved with *t*_½_ = 9.7 ± 1.8 s (Fig. 2). These results show that RNase H sequence preference contributed to PPT recognition, but even the poorest substrate of the three was cut ∼100-fold faster than PPT2, indicating the involvement of other factors.

PPT1 and CL4 substrates were cleaved too fast for reliable half-life estimation. Therefore, for cross-linked complexes of these two hybrids, we performed quenched-flow experiments in the 10-ms to 10-s time range as described previously (8). Both substrates showed biexponential decay, and the determined *t*_½_ values for the major reaction were 0.20 ± 0.01 and 0.18 ± 0.03 s for PPT1 and CL4, respectively (Fig. S7 and Table S1). Therefore, these substrates are cleaved at a very similar rate.

In our RNase H cleavage experiments, we observed gradual accumulation of the product until a plateau was reached when there was no more processable substrate available. This gradual reaction progress is in agreement with the fact that in the cross-linked complexes the substrate is not preorganized at the RNase H active site and for the RNA cleavage a conformational change is required. This change constitutes an energy barrier and occurs stochastically, resulting in gradual product generation.

Our previous results showed that the conformational change occurs mostly in the portion of the hybrid immediately upstream of the cleavage site (8). To verify whether this region of the substrate harbors the determinants of cleavage or protection, we introduced the PPT2 sequence between positions −7 and +2 of the otherwise random-sequence CL4 substrate, resulting in hybrid PPT2_2. This substrate was cleaved nearly as inefficiently as PPT2 (*t*_½_ = 13.6 ± 1.8 min), indicating that the part of the substrate that was located before and at the RNase H active site was important for PPT recognition (Fig. 2 and Figs. S5 and Fig. S6). In PPT2, this region comprised a stretch of adenines. We sought to determine whether it contributes to PPT protection. Substrates with a stretch of adenines or uridines in the RNA strand between positions −7 and −2 (6A, 6U, and 5U) were cleaved slowly with *t*_½_ = 2.7 ± 0.8 min for 6A, *t*_½_ = 6.5 ± 0.7 min for 5U, and *t*_½_ = 29.5 ± 3.4 s for 6U. These findings led us to conclude that RNase H cleavage immediately after a stretch of adenines or uridines was hindered. This explains the protection of the PPT body downstream from its own A-tract. Moreover, in the HIV-1 genome, the PPT sequence is preceded by the U-tract sequence. Our observation of poor RNase H cleavage after a U-tract would explain its function: it protects the PPT that is located downstream. These results are also consistent with the observation that the U-tract could be substituted with an A-tract without affecting (+)-strand initiation from the PPT (27).

We then tested whether the homopolymeric nature of A-tracts and U-tracts is important for PTT protection. We designed a series of substrates in which the homopolymeric nature of the nucleic acid was broken by introducing alternating adenines and uridines in the RNA strand. The introduction of six alternating adenines and uridines (UA) or three uridines followed by three adenines (3U) resulted in a similar *t*_½_ for both substrates (∼18 s; Fig. 2). This value was comparable with the random sequence with non-preferred residues in discriminating positions, indicating that the efficiency of hydrolysis was determined by the sequence consensus of RNase H cleavage rather than the particular properties of the A- or U-rich tracts. Therefore, higher resistance of the substrate to cleavage was conferred by homopolymeric stretches of more than three adenines or uridines.

Substrate 6A was still hydrolyzed 6-fold faster than PPT2. However, its variant that contained a guanine in position +1 (6A_G) was cleaved with a *t*_½_ of 13.6 ± 2.1 min, nearly as inefficiently as PPT2. Thus, the introduction of a guanine in position +1, combined with a stretch of adenines, which also resulted in non-preferred residues in all cleavage consensus positions, recapitulated the behavior of the PPT.

### Dynamic properties of homopolymeric tracts

**Figure 3.**
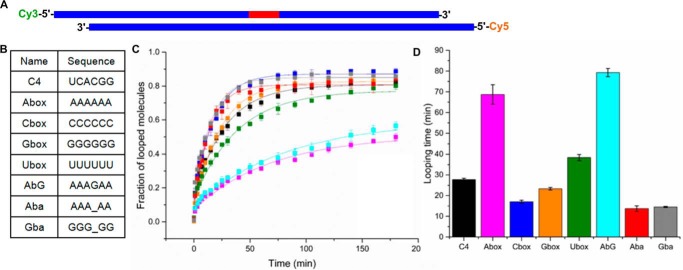
**Flexibility of RNA/DNA hybrids (looping assay).** *A*, schematic representation of the substrates that were used in the assay. *Blue* and *red bars* represent DNA and RNA, respectively. Fluorophores on 5′ termini of the strands are indicated. *B*, sequences of the 6-nt RNA fragments in the substrates. Abasic sites are indicated with *underscores. C*, looping of substrates with 6-bp RNA/DNA hybrid segments. Error bars represent S.D. of three independent measurements. C4, *black*; A box, *magenta*; C box, *blue*; G box, *orange*; U box, *green*; AbG, *cyan*; Aba, *red*; Gba, *gray. D*, looping times of the analyzed substrates.

Conformational changes in RNA/DNA are required for RNase H cleavage (5, 6, 8). We hypothesized that the dynamic properties of homopolymeric segments of the PPT RNA/DNA hybrids may be involved in protection of the PPT body. To test this possibility, we investigated the flexibility of such segments in DNA looping assays (28). A 6-bp RNA/DNA segment was introduced into an 80-bp dsDNA fragment with complementary overhangs and terminal fluorophores (Fig. 3, *A* and *B*). The closing time of the DNA circle, which depended on the flexibility of the inserted RNA/DNA fragment, was measured by monitoring single-molecule Förster resonance energy transfer (FRET) between the fluorophores. The results showed much higher rigidity of the poly(rA/dT) segment (*i.e.* slower loop formation) compared with either the random sequence or other homopolymeric sequences (Fig. 3, *C* and *D*). The AAAGAA sequence that corresponded to a fragment of the HIV-1 PPT was also rigid. This suggests that the rigidity of poly(rA/dT) protects it from RNase H cuts by prohibiting the conformational change that is required for RNase H cleavage.

**Figure 4.**
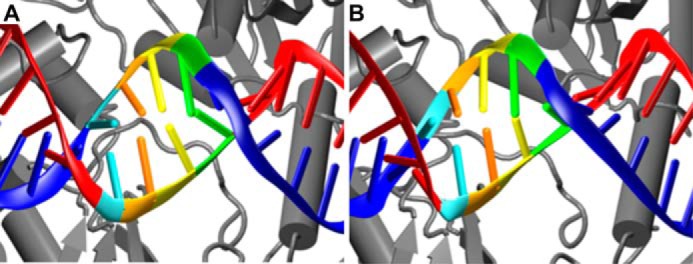
**Base-pair slippage of poly(rA/dT) tracts.** *A* and *B*, example of transient base-pair slippage observed in the molecular dynamics simulation of the PPT2 substrate. The protein is in *gray*. The RNA and DNA strands are in *red* and *blue*, respectively. The A-T base pairs are in color: −2 (*green*), −3 (*yellow*), −4 (*orange*), and −5 (*cyan*). *A*, structure before the simulation. *B*, shift in base pairing (slippage) that is observed when the catalytic interaction between the RNase H domain and PPT2 substrate is imposed.

**Figure 5.**
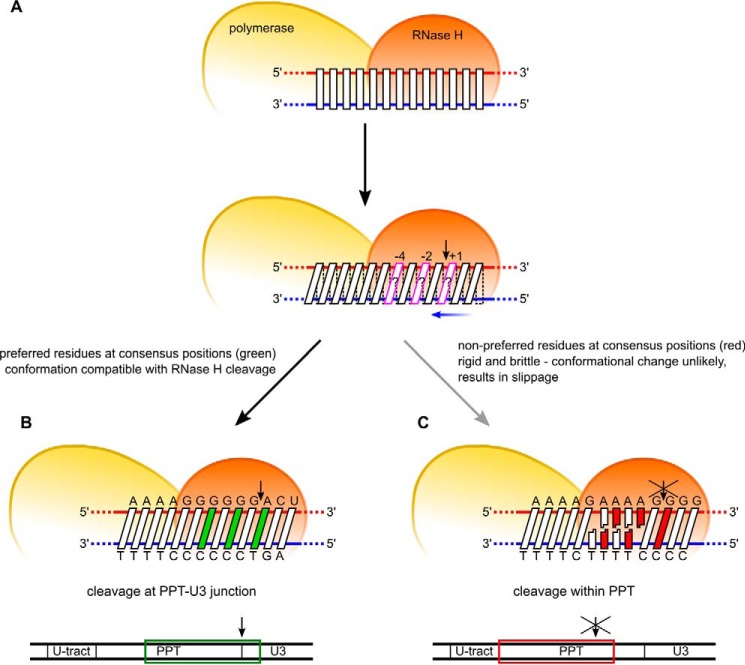
**Model of PPT recognition by HIV-1 RT.** *A*, two elements are involved in PPT recognition: sequence preference in positions −4, −2, and +1 (*magenta*) for cleavage by the RNase H domain and conformational changes of the substrate that are required for RNase H cleavage to occur. *B*, cleavage at the expected site (PPT-U3 junction) involves both preferred residues at the cleavage consensus positions (*green*) and the ability of the PPT sequence to undergo the conformational change without distortion. *C*, cleavage in the middle of the PPT body (A-tract) is inhibited by three elements: (i) non-preferred residues in the consensus positions (*red*), (ii) rigidity of the poly(rA/dT) sequence that makes the conformational change less likely (*gray arrow*), and (iii) deformations of the substrate (poly(A)-sequence slippage) when the deformation is enforced, leading to misalignment of the RNA at the RNase H active site. Ranges of PPT residues that are included in each schematic are indicated with *colored frames* at the *bottom*.

To explore this further, the PPT, poly(rA/dT), and poly(rU/dA) substrates were analyzed in 200–300-ns MD simulations (Table S2). The MD simulation setup was the same as in our previous work (8). The starting model for the simulations was a modified (Protein Data Bank code 4PQU) structure of HIV-1 RT bound to RNA/DNA in polymerase mode. We changed the sequence of the RNA/DNA to match the sequence of a particular substrate that was used in the biochemical experiments. To promote the catalytic interaction between the substrate and the RNase H domain, we imposed distance restraints based on the structures of substrate complexes of cellular RNases H1 (29, 30): (i) maximum distance of 7.2 Å between the C-α of Asp-443 at the heart of the active site and the phosphorus atom of the hydrolyzed phosphate of the RNA (between nt +1 and −1) and (ii) a pair of restraints between non-bridging oxygen of the phosphate group that was 2 bp from the scissile phosphate and C-α atoms of residues forming a critical phosphate-binding pocket element, Thr-473 (maximum distance of 5.8 Å) and Lys-476 (maximum distance of 5.3 Å). The simulations showed that the PPT1 substrate readily adopted a conformation whereby it catalytically interacted with the RNase H domain. The conformational change in the RNA/DNA was very similar to the one observed for random sequence substrate and described in detail previously (8). It mostly involved untwisting in the region that was located close to the RNase H domain, whereas the part of the hybrid bound by the polymerase domain was unchanged. When the PPT2 substrate was brought into a catalytic interaction with the RNase H domain, it exhibited pronounced deformations of helical geometry. These deformations were localized to the poly(rA/dT) stretch (−5 to −2) that interacted with the RNase H domain and mainly involved perturbations of base pairing, including excessive buckling of the base pairs and temporary shifts in the pairing register (*i.e.* base-pairing slippage; Fig. 4 and schematically shown in Fig. 5*C*). Behavior similar to PPT2 was observed in simulations of the 6A substrate complex (Table S2). The sequence slippage of poly(rA/dT) is very likely to lead to misalignment of the RNA strand at the RNase H active site and prevent cleavage. We assumed that the slippage was attributable to a combination of two effects: (i) the homopolymeric nature of the sequence and (ii) intrinsically weaker base pairing of A-T *versus* G-C. The latter was consistent with experiments in which non-hydrogen-bonding isosteres of cytosine that weaken base pairing were introduced into the G-tract of the PPT. This resulted in relocation of the cleavages further downstream (31). In light of our findings, this can be explained by the higher propensity of the modified G-tracts to undergo sequence slippage and the inability to align with the RNase H domain. To further support the hypothesis of poly(rA/dT) or poly(rU/dA) sequence slippage, UA and 3U substrates, in which purines alternate with pyrimidines and which are very unlikely to undergo slippage, were cleaved at rates that are expected based only on the RNase H sequence preference consensus (Fig. 2 and Figs. S5 and S6). In summary, two elements contribute to protection of the substrate downstream from the poly(rA/dT): (i) the rigidity of such a tract that prevents conformational changes in the nucleic acid that are required for RNase H cleavage and (ii) base-pair slippage when these conformational changes are enforced.

The simulations also suggested a potential mechanism of the sequence preference of the RNase H domain that was determined experimentally (see above) for base pair positions +1, −2, and −4 of the substrate. This mechanism is described in more detail in the supporting information. Briefly, our simulations imply that sequence recognition in position +1 relies on a specific contact between Arg-448 and the minor groove of the rA-dT/rU-dA base pair (Fig. S8). This interaction was much weaker or non-existent for the rG-dC/rC-dG pair because of steric and electrostatic hindrance from the amino group of guanine, thus disfavoring the rG-dC/rC-dG pair at this position. The sequence preference in position −2 can be explained by the structural properties of the substrate. In our simulations, the substrate- deforming interaction of the DNA strand with the phosphate-binding pocket of the RNase H domain causes base pair distortions for the −2 base pair. The preference for rG-dC/rC-dG in position −2 can thus be explained by the higher stability of this base pair over rA-dT/rU-dA. Lastly, no direct protein-nucleic acid interactions or substrate structural properties are observed in the experimental structures or our simulations that could explain sequence preference in the −4 position. However, there is a hydrogen bond between the Tyr-501 side-chain hydroxyl and phosphate between nucleotides −3 and −4 of the DNA strand and a van der Waals contact between the tyrosine aromatic ring and the ribose ring of nt −3. In our simulations, the stability of the H-bond strongly correlates with the pucker of the −3 nucleotide on the DNA strand. The C2′-endo region pucker is required for an ideal contact (Fig. S9a). When the base in position −4 of DNA is a thymine, steric clashes with its bulky methyl group prevent the C2′-endo sugar pucker of the −3 nucleotide (Fig. S9b), thus explaining why the −4 rA-dT (but not rU-dA) pair is disfavored in the substrate.

In our experiments, we also tested substrates that contained abasic sites. The introduction of an abasic site increases the flexibility of nucleic acids (32, 33), which was observed in our looping assay (Fig. 3). Molecular dynamics simulations of the 6A substrate that contained an abasic site revealed base-pair slippage and deformations that were even greater than for the parent duplex (Table S2). Moreover, in the cleavage assay, the 6A substrate with an abasic site at the −4 position (6A_a) was a very poor substrate for HIV-1 RNase H and was cleaved 4-fold more slowly than 6A (Fig. 2). The reduction of RNA/DNA rigidity by introducing an abasic site should promote RNase H cuts. However, it was offset by excessive slippage of the poly(rA/dT) sequence with an abasic site, leading to misalignment of the RNA at the RNase H active site. This confirms the importance of poly(rA/dT) sequence slippage in protecting the PPT body.

## Discussion

The precise mechanism by which the PPT primer is specifically generated by HIV-1 RT has remained elusive despite many years of extensive studies of this enzyme. The properties of PPT that serve for its recognition are unclear. In isolation, the PPT does not exhibit any special structural features (18) and is not inherently resistant to RNase H cleavage (10, 19). We developed a unique approach of enzymatic studies of chemically cross-linked HIV-1 RT–substrate complexes that allowed us to freeze the substrate-enzyme complex in particular configurations (8) (see supporting discussion for additional considerations for this approach). Consequently, we were able to monitor PPT cleavage independently of RNA/DNA binding and enzyme sliding events and at defined sites.

To explain PPT generation, one could presume that HIV-1 RT would have higher affinity for a certain part of the PPT sequence and/or its structural features (11, 31, 34). This would lead to the preferential association of the RT with the PPT RNA/DNA hybrid in a particular register to find the proper cleavage site at the PPT-U3 junction. HIV-1 RT is also known to slide on longer fragments of the nucleic acid (21). Therefore, another possibility is that during sliding the enzyme can stall in the proper cleavage register as a result of higher affinity for a particular sequence. Our results, however, demonstrate that these affinity-based mechanisms do not determine PPT generation as supported by two lines of evidence. First, we overcame the potential binding barrier by chemically cross-linking the substrate with the HIV-1 RT in a register that forced unfavorable cleavage within the PPT body (Fig. 1, substrate PPT2). The cleavage events were still very inefficient (Fig. 1*C*). Second, we measured the affinity of HIV-1 RT for the PPT substrates. We used two hybrids with recessed 3′ ends of the DNA strand. These ends would interact predominantly with the polymerase active site, positioning the RNase H active site either on the preferred cleavage site or within the PPT body, which should be protected. The dissociation constants for these two hybrids were very similar, showing that particular PPT binding registers did not influence HIV-1 RT–substrate interactions. Therefore, events within the stable complex are responsible for PPT generation.

Another element of the HIV-1 RT mechanism is flipping of the orientation of the enzyme on the substrate. It allows the protein to interact with an RNA primer hybridized to a DNA template either through the RNase H active site for its degradation or polymerase active site for its extension (16). The latter orientation is only observed for PPT primers, explaining their unique ability to prime the synthesis of (+)-DNA strand. Flipping could also contribute to PPT generation. RNase H orientation could be preferred for binding registers promoting proper PPT cleavage at the PPT-U3 junction, and only polymerase orientation could be present for registers that would result in cleavages in the middle of the PPT body. So far the flipping has been evaluated on a mature PPT primer or its versions with 3′ end extended by two RNA or DNA nucleotides (16). Other configurations, for example in the context of longer hybrids comprising the PPT (before its generation by RNase H) or for positions of RT that would potentially promote cleavages within the PPT body, have not been evaluated. Therefore, the contribution of flipping to the PPT protection has not been fully tested. Our data, however, demonstrate that the events within HIV-1 RT complexes that adopt the RNase H orientation already confer a mechanism of specific PPT recognition.

RNase H cleavage can occur concurrently with DNA polymerization with approximately every seven dNTPs added to the growing DNA chain (35). Later, polymerization-independent RNase H activity also occurs when HIV-1 RT associates with the fully synthesized RNA/DNA with a nicked RNA strand to complete its hydrolysis. The recognition of the PPT within the RT-substrate complex that we observed is consistent with both RNase H activities and in particular is compatible with the polymerization-dependent activity. HIV-1 RT does not dissociate from the substrate and presumably does not change its orientation (flip between polymerase and RNase H orientations) while it is processively polymerizing the DNA. During this process, within the RT-nucleic acid complex, the RNase H domain is presented with various sequences of translocating RNA/DNA substrate, including the PPT whose body must be protected.

Introduction of a covalent tether between HIV-1 RT and the RNA/DNA substrate should define the cleavage position 18 bp from the polymerase active site. However, in our experiments, we observed multiple cuts, particularly for the PPT2 and CL4 substrate complexes (Fig. 1*C* and Fig. S5). These observations were also confirmed in single-turnover kinetic experiments and are an important demonstration of the flexibility of the HIV-1 RT–nucleic acid complex (7). Various conformations have been observed in crystal structures of HIV-1 RT. We also observed conformational flexibility of the HIV-1 RT–hybrid complex in our MD simulations. Two elements of this flexibility are important for the location of the RNase H cuts: (i) the RNase H domain can change its position to reach various cleavage sites, and (ii) the RNA/DNA substrates can undergo conformational changes by overwinding and unwinding and thus allowing several phosphate groups to interact with the RNase H active site. This flexibility of the complex, combined with the large distance between the cross-link site and RNase H active site (which were located at the two ends of the complex), resulted in several cleavage sites. The frequency of these additional cuts was in agreement with the RNase H sequence preference consensus. For example, additional cleavages in the PPT1 substrate were much less efficient because only the cut at the PPT-U3 junction and not at adjacent sites met the sequence consensus. The PPT2 substrate was a very poor substrate in all registers around the expected cut 18 bp from the polymerase active site, so both of the observed cleavages were equally likely. In fact, at shorter times, upstream cleavage occurred before the expected cleavage (Fig. 1*C* and Fig. S5).

Several elements have been proposed to play a role in PPT recognition. RNase H sequence preference has been extensively studied and was found to be consistent with specific cleavages at the termini of the PPT (36). The geometries of the homopolymeric tracts that comprise the PPT and their junctions were also considered determinants of cleavage and protection (31). For example, one postulation was that the narrowed minor groove and extensive base stacking of poly(rA/dT) tracts determine their rigidity and resistance to RNase H cleavage (37). The structure of HIV-1 RT in complex with a PPT-containing RNA/DNA hybrid (Protein Data Bank code 1HYS) revealed shifted base pairing (“unzipping”) in the 5′ poly(rA/dT) tract (38). This distortion has been suggested to act as a binding determinant that interacts with the thumb domain to properly position HIV-1 RT on the PPT (39–41). Slippage of the substrate at the RNase H domain that we observed in our MD simulations and that was supported by the biochemical experiments resembles the unzipping of the poly(rA/dT) tract in the 1HYS structure. This is consistent with the notion that slippage of an rA-tract can occur. However, the 1HYS structure corresponds to the polymerase mode substrate conformation, and our MD simulations demonstrated that slippage is a consequence of conformational changes in the substrate that are required for RNase H cleavage rather than a pre-existing distortion.

In the present study, we experimentally verified the involvement of the above factors in PPT recognition, assessed their contribution, and combined them into one comprehensive model of the mechanism of PPT generation (Fig. 5). We propose that protection of the PPT body and generation of cuts at its termini result from a mechanism with two critical elements. The first element is the previously determined substrate sequence preference of the RNase H domain (22–26). The second and dominant element is a novel indirect readout of the poly(rA/dT) sequence, which is possible because of its rigidity and propensity to undergo base-pair slippage upon deformation. In fact, both elements of this mechanism rely on conformational changes in the substrate. For sequence preference, more efficient interactions between the RNase H domain and preferred nucleic acid sequence stabilize the substrate conformation that is required for RNase H cleavage. For an indirect readout of poly(rA/dT), conformational changes in the substrate are used to probe its rigidity and its dynamic properties (*i.e.* propensity to undergo slippage upon deformation). We suggest that the productive catalytic interaction and base pair distortion/slippage are two thermodynamically competing structural processes that are both induced by the protein-substrate interaction. In the PPT2, poly(rA/dT), and poly(rU/dA) substrates, the energy barrier of the base pair distortion/slippage would be lower than the energy barrier for productive binding at the RNase H domain. In other words, these substrates are rigid but also too brittle to be properly arranged for the chemical reaction. This was demonstrated by greatly reduced cleavage rates in the RNase H cleavage assays and structural distortions when forcing the catalytic interaction in the simulations. The opposite was true for the PPT1 substrate, which had a fast cleavage rate and no structural distortions in the simulations. This demonstrates another important component of our study, namely good agreement between the computational and experimental results.

Our results showed that a homopolymeric stretch of more than three adenines or uridines confers higher resistance of the substrate to RNase H cleavage. However, a combination of three uridines followed by three adenines in the 3U substrate only moderately affected downstream RNA cleavage in the RNase H cleavage assays. Interestingly, the uridine-adenine sequence is present at the U-tract–PPT junction, which could enable cleavage of the PPT downstream within the A-tract. However, for such a cleavage event to occur within the PPT, the +1 position would be occupied by an intervening guanine in the A-tract, which is a non-preferred residue according to the RNase H sequence consensus, thus leading to inefficient cleavage. Therefore, we propose that the intervening guanine in the A-tract evolved to protect the PPT body downstream from the U-tract–PPT junction. Our single-molecule FRET experiments showed that the introduction of a single G into the A-tract did not affect its rigidity or presumably its dynamic properties. Another interesting aspect to consider is the role of the stretch of six guanines in the PPT, which very likely serves two purposes. Stronger base pairing of the G-C pair increases stability of the duplex, preventing slippage and promoting RNase H cleavage downstream. Additionally, the presence of a stretch of guanines means that, for multiple registers of cleavage downstream from the PPT-U3 junction, the RNase H sequence preference consensus for positions −2 and −4 will match. Therefore, multiple cuts will be performed downstream from the PPT-U3 junction, thus clearing RNA away to make space for DNA polymerization from the PPT primer.

In summary, using a unique approach that combined enzymatic studies of chemically cross-linked substrate complexes of HIV-1 RT, MD simulations, and single-molecule experiments, we revealed the mechanism of PPT generation, which has remained elusive despite extensive studies of this clinically important enzyme. The present study provides a methodological and conceptual framework for further research that will lead to a better understanding of the intricate mechanisms of reverse transcription and intriguing and sophisticated enzymes that catalyze it: RTs.

## Experimental procedures

### RNase H activity assay for cross-linked complexes

Expression and purification of HIV-1 RT, chemical modification of DNA nucleotides, the cross-linking procedure, and purification of the cross-linked complexes were performed as described (8). The fraction of HIV-1 RT cross-linked with the nucleic acid in the final purified sample was determined based on densitometric quantification of Coomassie-stained acrylamide gels. Depending on the preparation, this fraction varied between 69 and 97%. The complexes comprised hybrid substrates PPT1 (RNA strand 5′-CCACUUUUUAACAGAAAAGGGGGGACUGGA-3′) and PPT2 (RNA strand 5′-CUUAGCCACUUCUUAAAAGAAAAGGGGGGA-3′). The fluorescent labels (Cy5 on 5′ end and fluorescein on the 3′ end) were introduced during solid-phase synthesis. The RNase H activity of the cross-linked complexes was assayed as described (8).

### RNase H activity assay for kinetic analysis

Samples that contained 60 nm cross-linked complexes that comprised RNA/DNA hybrids of various sequences with the 5′-terminal Cy5 label (Fig. 2*A*) were preincubated at 37 °C for 30 min in 20 mm Tris (pH 8.0), 100 mm NaCl, and 3 mg/ml heparin. The purpose of this step was to inhibit with heparin any activity originating from non-cross-linked enzyme. The samples representing time 0 (with no magnesium added) were also prepared from a premixed sample that was preincubated at 37 °C. The cleavage reaction was performed at 37 °C for 7.5 s, 15 s, 30 s, 1 min, 2 min, 4 min, 8 min, or 16 min in the presence of 8 mm MgCl_2_ and stopped by the addition of 40 mm EDTA. The reactions were performed in triplicate. Hydrolysis products were analyzed on 20% denaturing Tris borate-EDTA-urea polyacrylamide gels and visualized by fluorescence readout. Cleavage efficiency was measured based on densitometric quantification and is expressed (as a percentage) as the ratio of fluorescence intensity of the product to the sum for the product and substrate. Substrate 6A_a contained an R-spacer as an abasic site mimic.

### Kinetic data analysis

The pseudo-zero-order model for the reaction, analogous to radioactive decay, was fit globally for each substrate to three independent measurements at each time point using the optimization procedure that is contained in Origin 9.0.0 software (OriginLab, Northampton, MA). To improve algorithm stability, the data were not averaged, and consequently no weighting was used. For complexes PPT2, PPT2_2, 5U, 3U, 6A, 6A_a, and 6A_G, biexponential fitting did not converge, so the data were fit to a monoexponential model according to Equation 1. For the other complexes, both mono- and biexponential fitting (Equation 2) were performed, and the fitting accuracy was estimated by the *F* test and corresponding *p* value (Table S1). The *p* values were <10% for substrates CL4, CL6, PPT1, and 6U, so the biexponential model was used. For substrates CL5 and UA, the *p* values were >10%, so the exponential model was used. For biexponential decay, the half-life value, corresponding to the more populated cleavage event, is reported and was used for substrate comparisons,

(Eq. 1) $$I\left(t\right)=A_{0}\;\cdot \;\left[1-\exp\left(-t\;\cdot \;\ln\left(2\right)/t_{1/2}\right)\right]$$

(Eq. 2) $$I\left(t\right)=A_{0}\;\cdot \;\left[1-\exp\left(-t\;\cdot \;\ln\left(2\right)/t_{1/2}\right)\right]+\alpha_{0}\;\cdot \;\left[1-\exp\left(-t\;\cdot \;\ln\left(2\right)/\tau_{1/2}\right)\right]$$

where *A*_0_ and α_0_ reflect a population of “processable substrate” (or the plateau level), and *t*_½_ and τ_½_ (equal to ln 2/*k*) are the times required to process 50% of the substrate.

### Quenched-flow experiments

0.01–10-s time-scale reactions were prepared using an SFM-400 apparatus (BioLogic, Seyssinet-Pariset, France) in quenched-flow mode. Essentially, the experiment was performed as described previously (8), but the triplicate reactions were collected at time points of 10 ms, 20 ms, 40 ms, 80 ms, 160 ms, 320 ms, 640 ms, 1.28 s, 2.56 s, 5.12 s, and 10.24 s.

### Measurements of the affinity between HIV-1 RT and PPT substrates

Equilibrium binding constants were determined in a filter binding assay. DNA strands of substrates were radiolabeled with [γ-^33^P]ATP (Hartmann Analytic, Braunschweig, Germany) and T4 polynucleotide kinase (Fermentas). Mixtures of purified HIV-1 RT and DNA/RNA hybrids (50-μl total volume in 100 mm NaCl, 50 mm Tris-HCl (pH 8.0), 5 mm CaCl_2_, and 10 μg/ml BSA) containing constant concentration of oligonucleotide (50 pm) and varying concentrations of protein (0.25–20 nm) were incubated for 30 min at 25 °C. Reaction mixtures were filtered through a 0.22-μm nitrocellulose filter (Whatman) in a dot-blot apparatus (Bio-Rad). Each well was washed three times with 200 μl of binding buffer. Dried filters were exposed to a phosphorimaging screen overnight. Images were scanned on a Typhoon Trio+ Imager (GE Healthcare), and retained radioactivity was quantified using ImageQuant software (GE Healthcare). The data were fit with GraphPad software to a one-site binding equation,

(Eq. 3) $$B=B_{\max}\times c/\left(K_{d}+c\right)$$

where *c* is the protein concentration, *B*_max_ is the maximum binding and, *K_d_* is the dissociation constant.

### Molecular dynamics simulations

Molecular dynamics simulations were performed using the same method as described (8).

### Measurement of flexibility of nucleic acid substrates

A single-molecule DNA cyclization assay was previously developed to measure the flexibility of short dsDNA (less than 100 bp) at the single-molecule level (28). All of the DNA strands (90 nt) were prepared by the splint ligation of two synthesized DNA fragments (Integrated DNA Technologies and Future Synthesis) and purified in 14% Tris borate-EDTA-urea gel. After purification, complementary strands were annealed to make dsDNA by heating at 80–90 °C for 10 min followed by gradual cooling over 3 h. In each pair, one of the strands contained a 6-nt RNA fragment, yielding a 6-bp RNA/DNA hybrid upon annealing. The final DNA construct had a central double-stranded region (80 bp), which was the subject of the flexibility measurements, and a single-stranded overhang at each end (10 nt). At the 5′ ends of the overhangs, one strand contained Cy3, and the other contained Cy5 as a FRET pair, and they were designed to be complementary to each other to stabilize the DNA loop.

The prepared dsDNA was immobilized on a PEG-coated quartz slide via a NeutrAvidin-biotin interaction. Single-molecule imaging was performed in the buffered solution (10 mm Tris-HCl (pH 8.0), 10 mm or 1 m NaCl, 1% (w/v) d-glucose (Sigma-Aldrich), 150 units/ml glucose oxidase (Sigma-Aldrich), 2170 units/ml catalase (EMD Millipore), and 3 mm Trolox (Sigma-Aldrich)). Starting with a low-salt buffer (10 mm NaCl) in which most of the DNA did not make a loop, looping was initiated by infusing a high-salt buffer (1 m NaCl). Because looped and unlooped DNA molecules were distinguishable based on the corresponding high and low FRET signals, we were able to monitor looping kinetics as an increase in high FRET–state molecules over time. Through single-exponential fittings of the looping curve (looped fraction over time), the looping time could be obtained. We were able to fit all the looping curves with a single-exponential function (*R*^2^ ∼ 0.97). For slow looping constructs, the asymptotic value of looped fraction at long times decreases likely because unlooping becomes relatively more significant. The DNA looping time was used as an indicator of DNA flexibility. All of the measurements were performed over 3 h at ∼22 °C in triplicate.

## Author contributions

M. N., J. Š., and M. F. designed the research and wrote the paper. M. F. performed most of the biochemical experiments and analyzed the results. M. K. performed the molecular dynamics simulations and interpreted the results. J. P. performed the kinetic analysis of biochemical data. K. S. and A. G. performed substrate binding experiments. S. P. and T. H. designed and performed the DNA cyclization assay.

## Supplementary Material

Supporting Information

## Footnotes

3 The abbreviations used are:

HIV-1: human immunodeficiency virus type 1
RT: reverse transcriptase
PPT: polypurine tract
MD: molecular dynamics
nt: nucleotide(s).
