# Supplementary material for: Mechanism of polypurine tract primer generation by HIV-1 reverse transcriptase
Source: J Biol Chem. 2017 Nov 9;293(1):191–202. doi: 10.1074/jbc.M117.798256 (PMC5766924; doi:10.1074/jbc.M117.798256)
Supplement: Supporting Information [file supp_293_1_191__index.html]

Mechanism of polypurine tract primer generation by HIV-1 reverse transcriptase — Mechanism of polypurine tract primer generation by HIV-1 reverse transcriptase — PPT primer generation by HIV-1 RT — Supporting Information 

# Mechanism of polypurine tract primer generation by HIV-1 reverse transcriptase

## Supporting Information

- Supporting Information (.pdf, 3.0 MB) - Supporting Information
