# Supplementary material for: Mechanism of polypurine tract primer generation by HIV-1 reverse transcriptase
Source: J Biol Chem. 2017 Nov 9;293(1):191–202. doi: 10.1074/jbc.M117.798256 (PMC5766924; doi:10.1074/jbc.M117.798256)
Supplement: Supporting Information [file 10.1074_M117.798256_jbc.M117.798256-1.pdf]

Mechanism of polypurine tract primer generation by HIV-1 reverse transcriptase

Małgorzata Figiel, Miroslav Krepl, Sangwoo Park, Jarosław Poznański, Krzysztof Skowronek, Agnieszka Gołąb, Taekjip Ha, Jiří Šponer, and Marcin Nowotny

Supplementary Results
Supplementary Discussion
Supplementary Figures S1-S9
Supplementary Tables S1-S2

SUPPLEMENTARY RESULTS

RNase H sequence preference mechanism

Various MD simulations performed for this study (Table S2), provide indications for the sequence preference mechanism of HIV-1 RNase H domain, which is partially responsible for proper PPT generation. In the simulations, we have frequently observed a formation of direct H-bond interaction between the Arg448 side chain and the minor groove of the +1 pair (Fig. S8). The nature of this H-bond was different depending on the identity of the pair (rA/dT or rG/dC). When the pair was rA/dT, two very stable bifurcated H-bonds would form between the thymine O2 atom and the Arg448 side chain. However, when a GC pair is positioned here, we observed either no stable interaction or only a single transient H-bond with the cytosine O2 atom (Fig. S8B). The amino group of guanine appears to pose a steric and electrostatic hindrance for Arg448 interaction with the minor groove. Further simulation tests confirmed that the result is the same when the base pair is inverted (rU/dA or rC/dG) except that then the arginine was also transiently interacting with N3 atom of the adenine. Thus, inversion of the polarity of the base pair does not affect the interaction.

The simulations strongly suggested that the catalytic interaction between the substrate and RNase H domain introduces certain structural stress into the substrate. The severity of this stress is sequence dependent, however, what was universally observed is that the largest disruptions of the base pairing occurred for the -2 base pair. Further, the structural distortions of the upstream regions of the helix often started first at this base pair. This is likely due to its proximity to the phosphate-binding pocket which requires the helix to unwind here. Therefore, the experimental preference for the rG/dC or rC/dG base pairs at the -2 position could be explained by the higher thermodynamic stability of the rG/dC or rC/dG base pairs over the rA/dT or rU/dA. Earlier studies suggested that a preference at position -2 may rely on a base-specific minor groove interaction with the Gln475 side chain (36). While we indeed observed this interaction in the simulations, it did not seem to have a clear base pair preference.

The next sequence preference site is at the -4 base pair where the adenine is disfavored in the RNA strand. This equals to the thymine being disfavored on the DNA strand. There is no direct interaction between the protein and the -4 bases. However, there is a hydrogen bond between Tyr501 side chain hydroxyl and the phosphate between nucleotides -3 and -4 of the DNA strand. There is also a vdW contact between the tyrosine aromatic ring and the -3 DNA strand sugar ring. What we noticed in simulations is that the stability of the h-bond strongly correlates with the pucker of the -3 nucleotide on the DNA strand. Specifically, a C2'-endo region pucker is required for ideal contact (Fig. S9A). It has been suggested earlier that a sugar pucker of a DNA nucleotide is lowered in case the following nucleotide base is a thymine (42). This is because of a steric repulsion between the thymine methyl group and the atoms of the sequentially preceding sugar ring. This effect is well reproduced in the simulations where the substrates with thymine as the -4 nucleotide produce vastly different sugar pucker populations in the -3 nucleotide than those with non-thymine nucleotide (Fig. S9B). Therefore, the simulations suggest that the experimental preference of the -4 base pair not to be an A-T can be explained in the following fashion: the presence of thymine as a -4 base likely stabilizes lower sugar pucker of the -3 deoxyribose which in turn may destabilize the important interactions with the Tyr501 side chain.

SUPPLEMENTARY DISCUSSION

Disulphide cross-linking as a method for capturing native complex structures

The thiol-based cross-linking approach that we employed traps mechanistically relevant states of nucleic acid enzymes. This is supported by several lines of evidence. In a related manuscript (8), we characterized chemically cross-linked HIV-1 RT–substrate complexes in more detail. We tested the specificity of this approach and showed that the covalent tether can specifically form for pairs of thiol-modified protein/nucleic acid residues that are located sufficiently close to each other in the structure of the complex. Thus, this approach stabilizes native conformations. Moreover, crystal structures were solved for HIV-1 RT in complex with analogous dsDNA substrates without cross-linking (2HMI) (43) and with the Q258C cross-link (1RTD) (4). These two structures are essentially identical, confirming that the cross-link captures a native structure. For RNA/DNA substrates, a crystal structure of a complex that is cross-linked using exactly the same approach as the one applied in this work (i.e., Q258C substitution combined with modification of the sixth base from the 3′ end of the primer) is available (4PQU) (6). In fact, we used it as a starting point in our MD simulations, which was possible because this structural model was very stable in these calculations. The 4PQU structure is in a fully productive polymerase mode, including the incoming nucleotide and divalent metal ions that are bound at the active site. In a related manuscript (8), we used two different methods and showed that the cross-linked complexes indeed bind the incoming cognate nucleotide specifically and with an affinity similar to the free enzyme. This confirms the proper organization of the polymerase active site, including orientation of the template and primer end. RNase H activity is also preserved in the cross-linked complexes. Finally, the cross-linking methodology has been used to determine crystal structures of multiple protein–nucleic acid complexes of important enzymes, including glycosylases (44-47).

SUPPLEMENTARY FIGURES

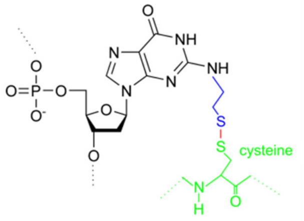

Figure S1. Schematic of the cross-linking chemistry. The two-carbon linker with a thiol group (blue) is tethered to the N2 atom of the guanine base. The disulfide bond that forms between the thiol group connected to the base and a cysteine residue (green) is shown in red.

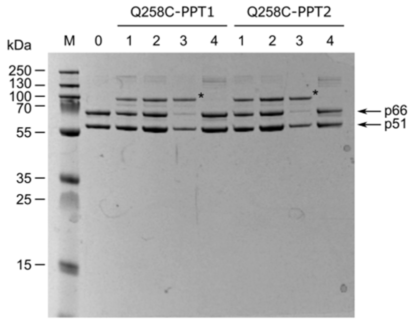

Figure S2. Purification of cross-linked complexes. M, molecular weight marker; 0, HIV-1 RT Q258C; 1, cross-linked sample; 2, nickel column elution; 3, heparin column flow-through; 4, heparin column elution. The band that corresponds to the cross-linked product is indicated with an asterisk.

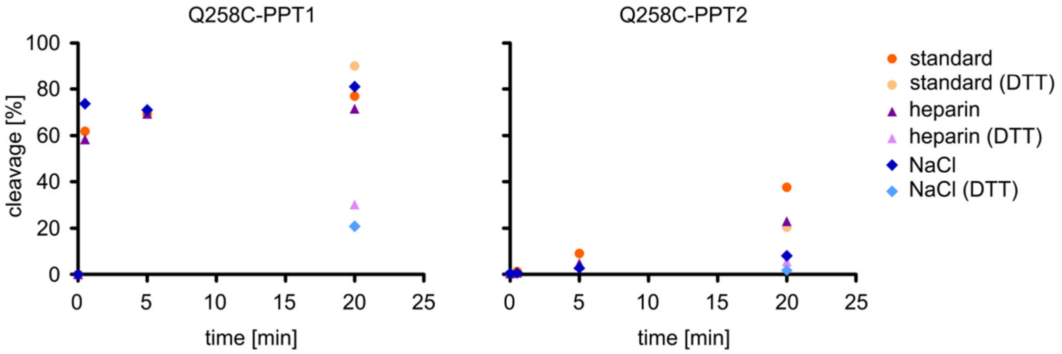

Figure S3. RNase H cleavage within cross-linked complexes with PPT substrates. Intensities of the bands from gels shown in Fig. 1c were quantified by densitometry and the extent of cleavage was expressed as the ratio of the fluorescence intensity of the product band divided by the total fluorescence in the lane. Results from reactions with samples pre-treated with 20 mM DTT are shown in light colors and indicated (DTT). Reactions were performed in a standard buffer containing 100 mM NaCl and no heparin, in the presence of heparin or in the presence of 0.5 M NaCl.

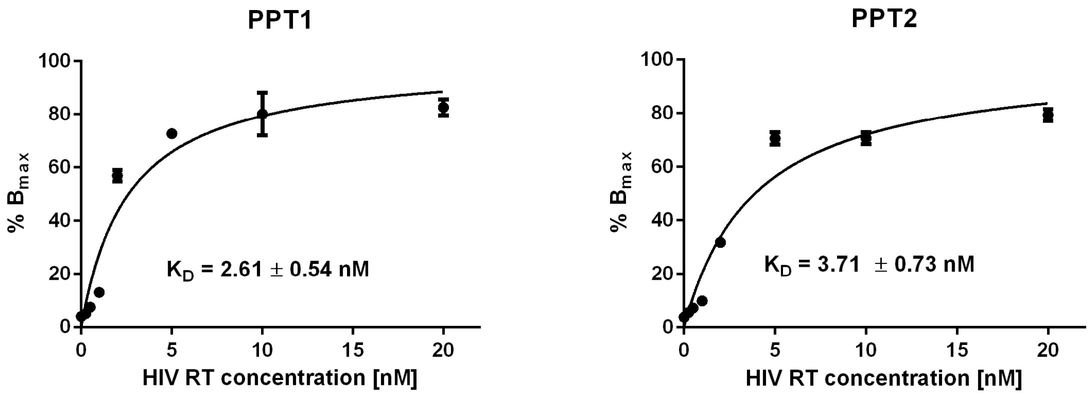

Figure S4. Affinity of wildtype HIV-1 RT for hybrid substrates measured by nitrocellulose filter binding assay. Bars indicate the standard deviation of three measurements.

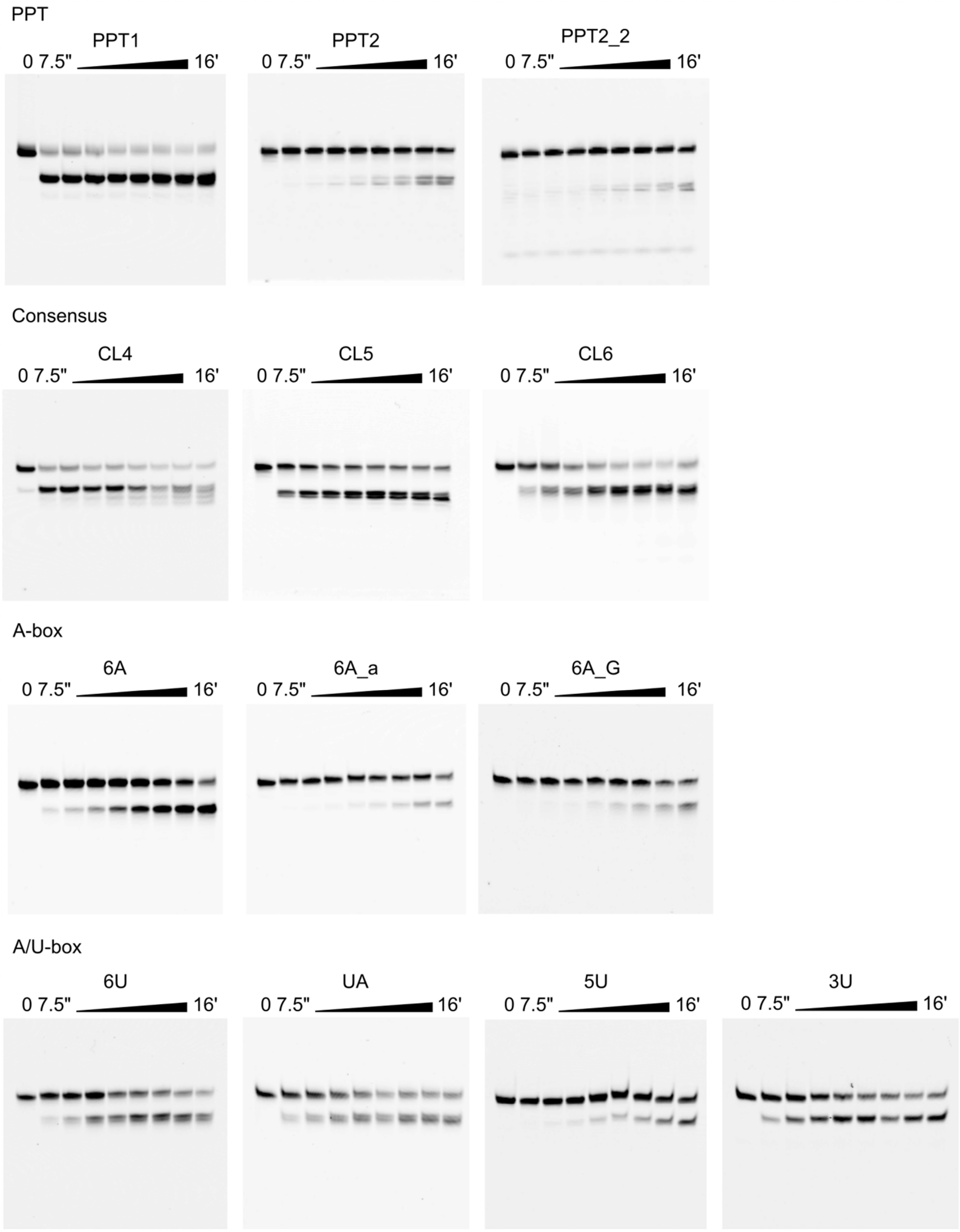

Figure S5. RNase H cleavage of different substrates cross-linked with HIV-1 RT. The reactions were performed at 37°C for 7.5 s to 16 min and stopped by the addition of 40 mM EDTA. Samples were analyzed on 20% TBE-urea denaturing gels. Samples from one of three independent experiments are shown. Quantification of the data is shown in Fig 2b.

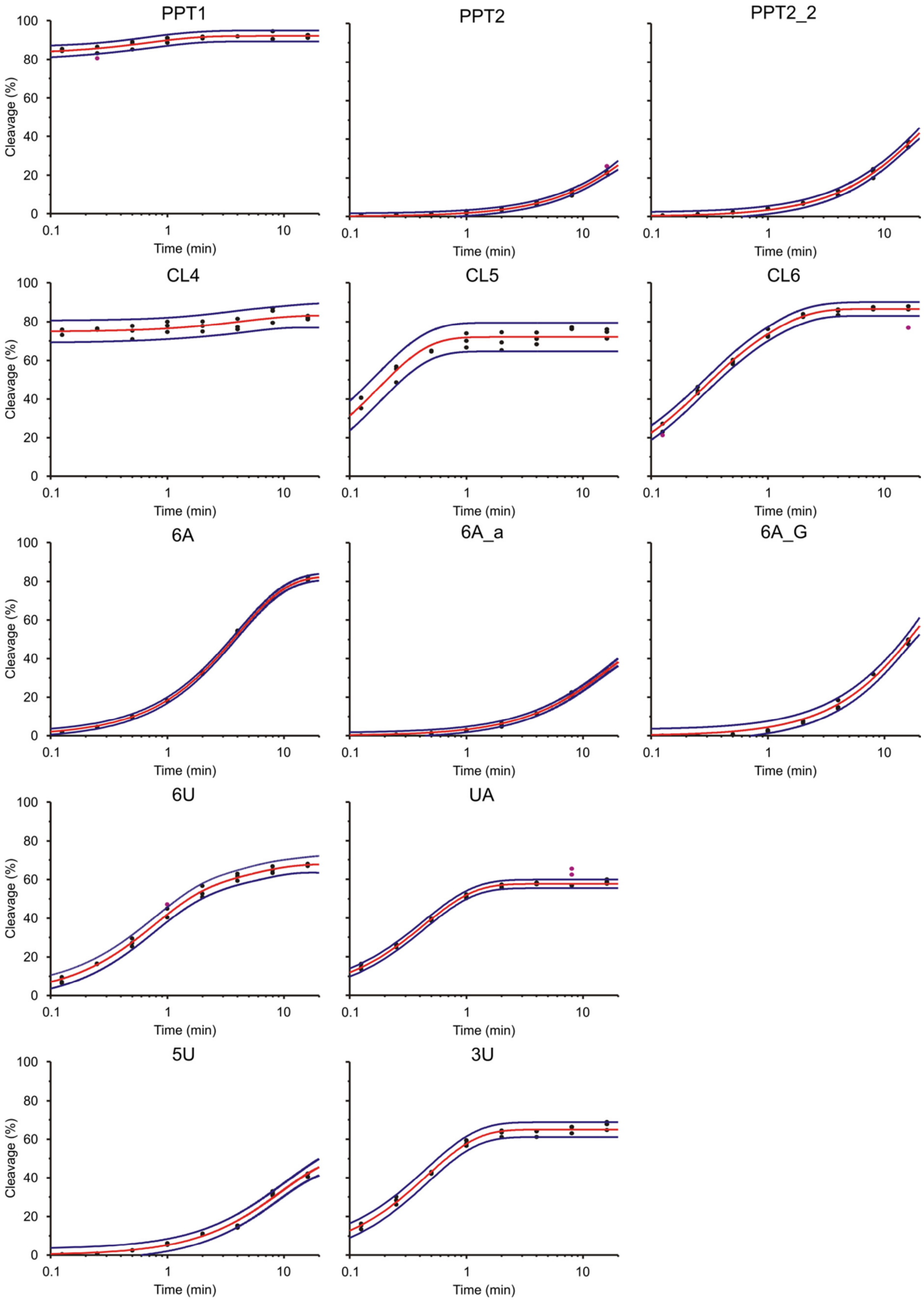

Figure S6. Fitting of the cleavage time-courses. Intensities of the bands from gels shown in Fig. S5 were quantified by densitometry and the extent of cleavage was expressed as the ratio of the fluorescence intensity of the product band divided by the total fluorescence in the lane. Global fitting of the data was performed to a pseudo-zero-order model of the reaction, analogous to radioactive

decay. Data for each substrate were fitted globally to three independent measurements of each time-point. The red line is the fit to the data and blue lines represent the 95% confidence limits for prediction band. Outlier measurements from prediction band that were removed from analysis are shown in magenta. The resulting half-life values calculated from the fit are given in Table S1 and Fig. 2.

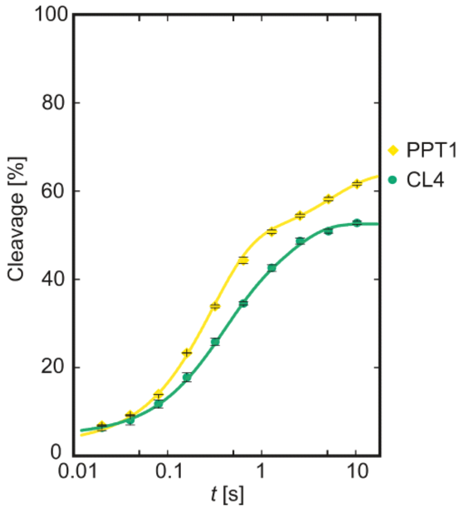

Figure S7. Quenched-flow experiment for CL4 and PPT1 complexes. RNase H cleavage within cross-linked complexes between HIV-1 RT and CL4 (green) and PPT1 (yellow) on a 0.01-10 s time-scale. The reactions were performed using a quenched-flow apparatus. The cleaved fraction of the substrate was quantified by densitometry. Data from three independent experiments were averaged and plotted for each time-point. The error bars represent standard deviation.

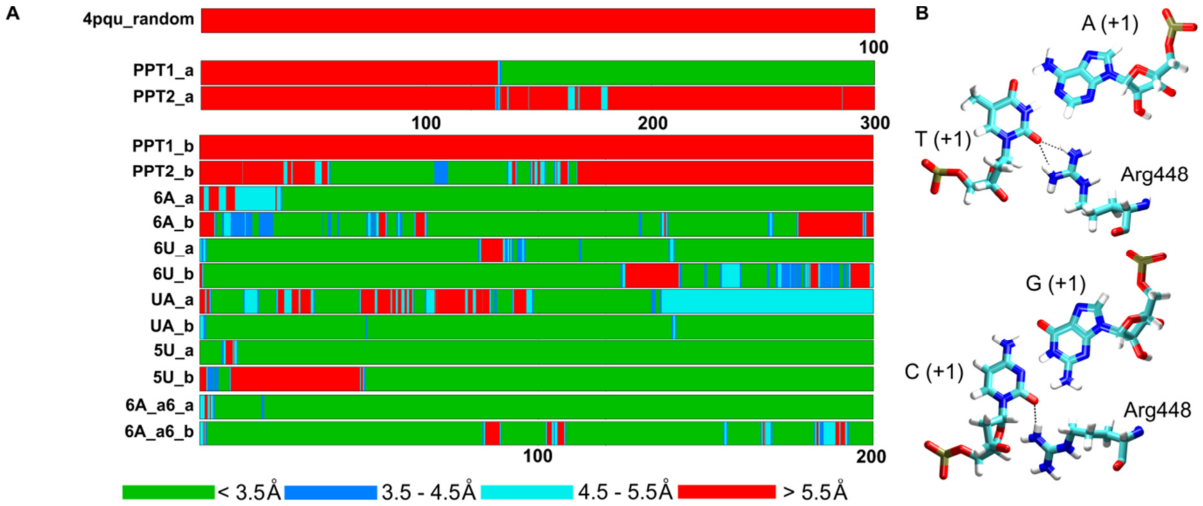

Figure S8. Mechanism of sequence preference at position +1. (A) Simulation time development [in ns] of the shortest heavy atom distance between Arg448 side chain nitrogens and O2 atom of the +1 base pair. **(B)** An Arg448 h-bond interaction in the minor groove with either the rA/dT (top) or rG/dC (bottom) +1 base pair. The PPT2 substrate contained rG/dC base pair at the +1 position while the other substrates had rA/dT. Despite the fact that the simulations started from initial structures with the Arg448 positioned far away from the +1 base pair, a stable h-bond interaction spontaneously formed in all simulations with the +1 rA/dT base pair except the second PPT1 simulation. In contrast, in the first PPT2 simulation (with rG/dC base pair), no equivalent interaction was formed despite that the residues came few times close to each other. In the second PPT2 simulation, a weaker alternative h-bond (shown in (B), bottom) was formed in part of the trajectory.

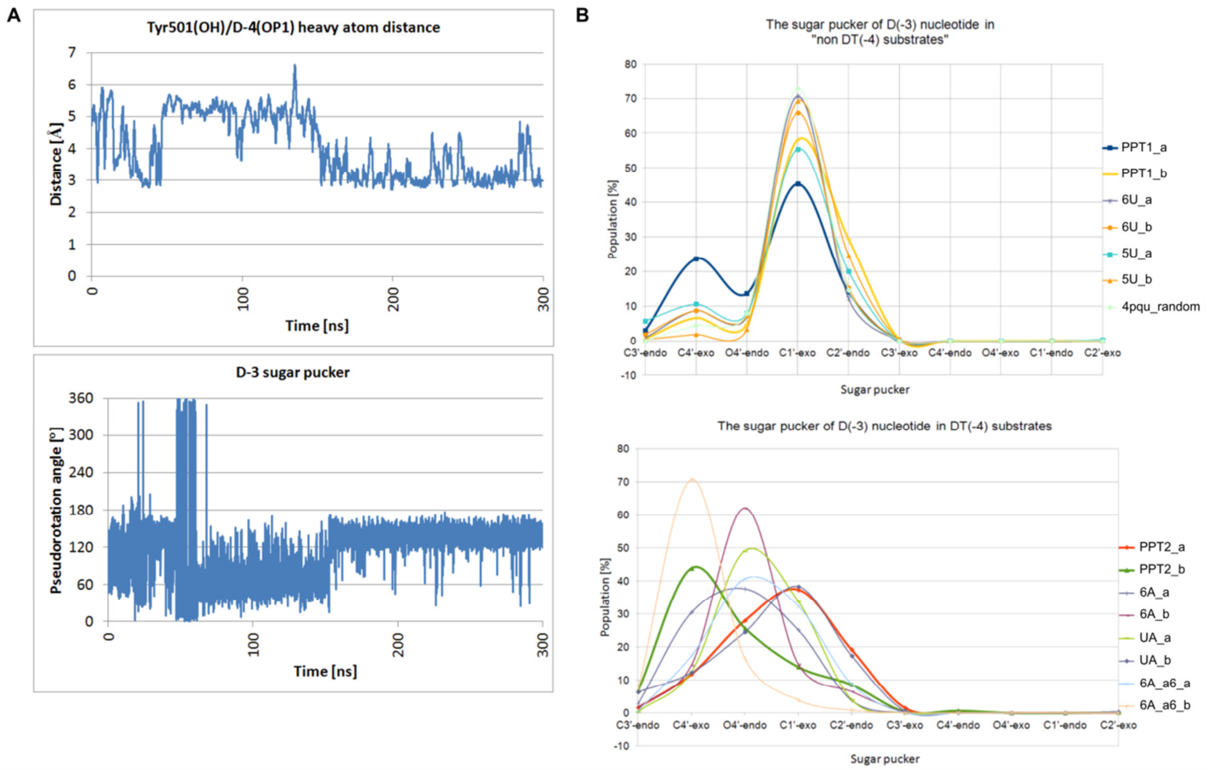

Figure S9. Mechanism of sequence preference at position -4. (A) An example of typical simulation time development of the Tyr501(OH)/D-4(OP1) heavy atom distance (top) and the sugar pucker pseudorotation angle of the D-3 nucleotide (bottom). The “D-4” and "D-3" nucleotide refer to the DNA nucleotides of the -4 and -3 base pair, respectively, regardless of the identity of the base. **(B)** The sugar pucker populations of the D-3 nucleotide in simulations where the D-4 nucleotide is not thymine (top) and in simulations where the D-4 nucleotide is thymine (bottom). The simulations not having T at the D-4 position show well separated (and converged) C2'-endo/C3'-endo region division while those with D-4 thymine show generally lower, non-converged pucker values.

Table S1. Parameters of fitting of the time-course data shown in Figure S6 and S7. Data were fit with exponential and bi-exponential model (see Experimental Procedures). Amplitude (A, α) and half-life ($T_{1/2}$, $\tau_{½}$) values are given with errors in parentheses. The significance of the minor reaction was assessed according to the F-test and p-value. Bi-exponential model was selected only for those fits that resulted in p-value below 10%. Parameters of selected models are shown in bold.

	Exponential		Bi-exponential					
			Major reaction		Minor reaction		Statistics	
	A [%]	$T_{½}$ [s]	A [%]	$T_{½}$ [s]	α [%]	$\tau_{½}$ [s]	F (n1,n2)	p
**Manual experiments (7.5 s – 16 min)**								
CL4	78 (1)	n.d.	**76 (1)**	**n.d.**	**7 (3)**	**330 (290)**	2.23 (18,20)	**0.047**
CL5	**72 (1)**	**7.0 (0.4)**	67 (3)	6.2 (0.6)	9 (3)	140 (120)	1.37 (22,24)	0.232
CL6	85 (1)	16.2 (0.7)	**52 (12)**	**9.7 (1.8)**	**35 (12)**	**39 (11)**	2.41 (22,24)	**0.021**
PPT1	90 (1)	n.d.	**83 (1)**	**n.d.**	**9 (1)**	**29 (8)**	4.08 (19,20)	**0.002**
PPT2	55 (11)	1250 (320)	n.c.					
PPT2_2	67 (6)	810 (110)	n.c.					
6A	80 (41)	159 (46)	n.c.					
6U	64 (1)	38.6 (1.8)	**51 (6)**	**29.5 (3.4)**	**17 (5)**	**180 (90)**	2.06 (22,24)	**0.047**
5U	52 (3)	392 (39)	n.c.					
3U	63 (2)	18.0 (0.8)	n.c.					
UA	**58 (0)**	**18.0 (0.4)**	56 (1)	17.3 (0.5)	4 (6)	600 (2000)	1.3 (22,20)	0.280
6A_a	**54 (3)**	**675 (58)**	n.c.					
6A_G	**89 (10)**	**820 (130)**	n.c					
**Quenched-flow (10 ms – 10 s)**								
CL4	51 (1)	0.43 (0.02)	**27 (4)**	**0.18 (0.03)**	**25 (4)**	**0.94 (0.15)**	3.78 (25,27)	**<0.001**
PPT1	57 (1)	0.30 (0.02)	**47 (1)**	**0.20 (0.01)**	**16 (1)**	**4.1 (0.5)**	31 (25,27)	**<0.001**

n.c. – not converged,
n.d. – value too small to be precisely determined

Table S2. List of simulations.

Simulation name[a]	Sequence (RNA)	Length (ns)	DNA/RNA substrate stability[b]
PPT1_a	PPT1	300	++
PPT1_b	PPT1	200	++
PPT2_a	PPT2	300	-
PPT2_b	PPT2	200	+
Cl4_6A_a	Cl4_6A	200	-
Cl4_6A_b	Cl4_6A	200	-
Cl4_6U_a	Cl4_6U	200	+
Cl4_6U_b	Cl4_6U	200	+
Cl4_UA_a	Cl4_UA	200	+
Cl4_UA_b	Cl4_UA	200	+
Cl4_6A_abasic_R-6_a	Cl4_6A with abasic site in position 6	200	-
Cl4_6A_abasic_R-6_b	Cl4_6A with abasic site in position 6	200	-
Cl4_5U_a	Cl4_5U	200	+
Cl4_5U_b	Cl4_5U	200	+

[a] To verify reproducibility of the results, two independent simulations were performed for all the systems (marked as "name_a" and "name_b" in the table). Both simulations usually provided mutually very consistent results. [b] Simplified assessment of the DNA/RNA substrate simulation stability: "++" are substrates where we observed no base pair slippage or distortions; "+" are substrates with moderate base pair distortions but no base-pair slippage; "-" are substrates with large base pair distortions and/or slippage.

REFERENCES

42. Tolstorukov, M. Y., Ivanov, V. I., Malenkov, G. G., Jernigan, R. L., and Zhurkin, V. B. (2001) Sequence-dependent B <-> A transition in DNA evaluated with dimeric and trimeric scales. *Biophys. J.* **81**, 3409-3421
43. Ding, J. P., Das, K., Hsiou, Y., Sarafianos, S. G., Clark, A. D., Jacobo-Molina, A., Tantillo, C., Hughes, S. H., and Arnold, E. (1998) Structure and functional implications of the polymerase active site region in a complex of HIV-1 RT with a double-stranded DNA template-primer and an antibody Fab fragment at 2.8 angstrom resolution. *J. Mol. Biol.* **284**, 1095-1111
44. Banerjee, A., Santos, W. L., and Verdine, G. L. (2006) Structure of a DNA glycosylase searching for lesions. *Science* **311**, 1153-1157
45. Bowman, B. R., Lee, S., Wang, S. Y., and Verdine, G. L. (2010) Structure of Escherichia coli AlkA in Complex with Undamaged DNA. *J. Biol. Chem.* **285**, 35783-35791
46. Fromme, J. C., Banerjee, A., Huang, S. J., and Verdine, G. L. (2004) Structural basis for removal of adenine mispaired with 8-oxoguanine by MutY adenine DNA glycosylase. *Nature* **427**, 652-656
47. Qi, Y., Spong, M. C., Nam, K., Banerjee, A., Jiralerspong, S., Karplus, M., and Verdine, G. L. (2009) Encounter and extrusion of an intrahelical lesion by a DNA repair enzyme. *Nature* **462**, 762-766
